# New insights into the stress response mechanisms of stress-resistant *Listeria monocytogenes* via multi-omics and cell morphological changes

**DOI:** 10.1080/22221751.2025.2564319

**Published:** 2025-09-19

**Authors:** Hyunhee Hong, Eiseul Kim, Seung-Min Yang, Min-Cheol Lim, Hyun Jung Kim, Liping Yang, Hae-Yeong Kim, Si Hong Park

**Affiliations:** a Department of Food Science and Technology, Oregon State University, Corvallis, OR, USA; b Korea Food Research Institute, Wanju-gun, Republic of Korea; c Department of Chemistry, Oregon State University, Corvallis, OR, USA; d Institute of Life Sciences & Resources and Department of Food Science and Biotechnology, Kyung Hee University, Yongin, Republic of Korea

**Keywords:** *Listeria monocytogenes*, stress resistance, RNAseq, proteomics, cell morphology, stress-related biomarkers

## Abstract

*Listeria monocytogenes* (*Lm*) can persist in stressful conditions such as low pH, temperature, and salt concentration. Omics approaches enable the investigation of biomolecular features at both the transcript and protein levels. This study aimed to understand stress response mechanisms by analyzing transcript and protein levels of stress-resistant *L. monocytogenes* (SR-*Lm*) under multiple stress conditions. RNA sequencing was performed at 4, 8, 12, 24, and 48 h growth under a multiple stress condition (pH 3 + 1 °C + 5% salt) and RT-qPCR was also conducted. Gene expression analysis revealed upregulation of the *yebS*, *tsx, secA, gap, gtfA*, along with several stress-related genes (*cspB*, *cspD*, *spxA*, and *cysK*) during 48 h. Protein expression profiles of SR-*Lm* were conducted under (1) 5% salt+1 °C, (2) pH 3 + 1 °C, and (3) pH 3 + 1 °C + 5% salt conditions at 4 and 12 h. Some proteins including Gap, Eno, Citz, and PurM were upregulated in both analyses, potentially associated with stress-related biomarkers. KEGG pathway analysis was performed using proteomic data to predict stress response mechanisms including chaperone, glycolysis, and glutamate decarboxylase system (GAD). Cell morphology under stress and normal conditions was observed, with some elongated, thin, and bumpy cell shapes under acid and multiple stress conditions. This study provides new insights into the stress response of SR-*Lm*, enhancing the understanding of how *Lm* survives in adverse food processing and storage environments.

## Introduction

*Listeria monocytogenes (LM)* causes listeriosis, which is found in soil, water, dairy products, ready-to-eat (RTE) foods, meats, and vegetables. This illness leads to approximately 1,600 hospitalizations and 260 deaths annually in the United States [1]. In 2020, an outbreak due to contaminated enoki mushrooms transported from several suppliers led to 31 hospitalizations and 4 deaths [1,2]. In 2018, the estimated cost of *Listeria* outbreaks in a restaurant was $4,748–$2,915,601 [3]. The overall economic burden due to listeriosis in 2023 was estimated at $4 billion [4]. *Lm* can survive in acidic, low temperature, and high salt environments in foods and processing [5,6]. According to a previous study, *Listeria* species under stress conditions were categorized into stress-resistant and stress-sensitive strains, with a difference in viable cell counts (approximately 4 log CFU/mL) [7,8]. The survival of resistant *Listeria* strains presents a significant challenge in food safety. This concern has prompted researchers to investigate the pathogen’s resistance mechanisms and to characterize its stress-related genetic factors. To understand the stress mechanisms at the molecular level, omics approaches have been utilized to study gene expression and protein function.

Transcriptomic analysis is used to study entire gene expression levels including mRNA. RNA sequencing (RNAseq) was conducted to analyze *Lm* with and without adaptation to 4% sodium lactate, an antimicrobial used in meat products [9]. This previous study suggested that adaptation to sodium lactate involves the ATP-binding cassette (ABC) transporter and the phosphotransferase system (PTS). Similarly, the gene expression levels of *Lm* under pulse magnetic treatment were altered, particularly affecting carbohydrate metabolism, the two-component regulatory system, and ABC transporters. These changes suggested potential stress mechanisms by highlighting the bacteria's response to pulse magnetic treatment [10]. Proteomic analysis provides insights into the protein characteristics and functions of *Lm* under specific environmental conditions. It also reveals how the pathogen adapts its metabolism to survive in diverse environments [11]. Proteomic studies of *Lm* under different growth temperatures (10–42 °C) have shown that heat and cold shock proteins were significantly altered with temperature variations [12]. Another study reported that proteomic analysis of *Lm* exposed to linalool (an antibacterial agent) showed significant changes in proteins associated with cell walls, nucleoids, and ribosomes [13]. These proteomic studies demonstrate effective omics methodologies for investigating stress response mechanisms in *Lm*.

*Listeria* species have been classified into stress-resistant and stress-sensitive groups [7,8]. However, research on the combined transcriptomic and proteomic responses of SR-*Lm* under stress conditions remains limited. Given these previous findings, we first performed transcriptomic (RNAseq) and protein quantification to identify the stress response mechanisms of SR-*Lm*. This study aimed to compare the transcript and protein levels of SR-*Lm* under stress conditions with those under normal growth conditions. By comparing transcripts and protein profiles and expression levels, we sought to establish stress-related genes and identify the mechanisms underlying stress responses and cell morphology changes under the stress condition.

## Materials and methods

### Bacterial strains and stress resistance tests

The SR-*Lm* BL42 isolated from food was selected and stress resistance tests were performed according to previous studies [7,8] under normal (control) and multiple stress condition (pH 3 + 1 °C + 5% salt) at 4, 8, 12, 24, and 48 h. The death curves of SR-*Lm* under normal (37 °C) and stress conditions were depicted, while each sample was collected at five different time points for RNA extractions. For proteomic analysis, the stress resistance tests were conducted under three different conditions: (1) 5% salt+1 °C, (2) pH 3 + 1 °C, and (3) pH 3 + 1 °C + 5% salt (multiple stress) for 12 h. The low temperature (1 °C) was set as the default in this study, considering real-world food conditions (e.g. refrigerated foods) associated with *Listeria* outbreaks [5,6]. Under the normal condition (37 °C, control), viable cells were plated on tryptic soy agar plates (Difco, Becton Dickinson, Sparks, MD, USA) and enumerated after 4 and 12 h, while 40 mL samples after centrifugation were collected for protein extraction. All experiments were performed in triplicate.

### RNAseq

Total RNA was extracted at 4, 8, 12, 24, and 48 h under normal and stress conditions using a RNeasy Micro Kit (Qiagen, Hilden, Germany) according to the manufacturer’s instructions. Three independent biological replicates were performed for each condition and pooled. The RNA concentration was assessed with a Qubit 4 Fluorometer (Thermo Fisher Scientific, Waltham, MA, USA) and diluted to 20 ng/μL for library preparation. RNAseq libraries were prepared using an Illumina-stranded total RNA prep kit with rRNA depletion and Illumina DNA/RNA UD indexes Set A (Illumina, San Diego, CA, USA) following the manufacturer's instructions. The library quality was confirmed using a bioanalyzer (Agilent 2100 Bioanalyzer, Santa Clara, CA, USA), and sequencing was performed on a NextSeq 2000 system (Illumina) to generate 150 bp paired-end reads at the Center for Quantitative Life Science at Oregon State University. Raw sequence reads were trimmed using the Sickle master tool and all paired reads were aligned with the Splicing Transcripts Alignment to a Reference (STAR 2.7) software (https://github.com/alexdobin/STAR) using the reference *Lm* strain ATCC19115 from the National Center for Biotechnology Information (NCBI). Compressed Sequence Alignment Map Format (SAM) files were processed on StringTie to predict the gene expression levels (i.e. transcripts per million; TPM) at https://github.com/gpertea/stringtie. Pseudocount was used, adding 1 to all TPM values to avoid issues with zero values. The log_2_ fold changes (logFC) of TPM in normal and stress conditions were calculated (stress TPM/normal TPM) at each time point. The cutoff of upregulated and downregulated genes was set at ±1 logFC.

### Reverse transcription-quantitative PCR (RT-qPCR) assay

The RT-qPCR assay was performed according to the previous studies [9]. The *gyrA* gene, which showed stable expression levels in the RNAseq results, was used as the reference gene (internal control). Genes identified as up- or down-regulated were selected to conduct RT-qPCR assay at five time points using an iTaq™ Universal SYBR® Green One-Step Kit (BioRad, Hercules, CA, USA). The reaction mixture was as follows: 5 μL of iTaq universal SYBR Green reaction mix (2×), 0.125 μL of iScript reverse transcriptase, 0.3 μL each of forward and reverse primer (300 nМ), 1 μL of total RNA (20 ng), and 3.275 μL of nuclease-free water, making a total volume of 10 μL. An Applied Biosystem 7500 (Thermo Fisher Scientific) was used for the thermal cycling: 35 cycles at 50 °C for 10 min, 95 °C for 1 min, and 35 cycles at 95 °C for 15 s and 60 °C for 30 s (melting curve 65–95 °C). The 2^-ΔΔCT^ method was used to analyze the relative gene expressions [14].

### Shotgun proteomic analysis

The grown cultures collected at 4 and 12 h were processed to extract proteins using a B-PER Bacterial Protein Extraction Reagent (Thermo Fisher Scientific). Trypsin-digested peptide samples were analyzed using an Orbitrap Fusion Lumos mass spectrometer with a Nano ESI source (Thermo Fisher Scientific), coupled with a Waters Acquity M-class UPLC system (Waters, Milford, MA, USA). Peptides were desalted and trapped on a nanoAcquity UPLC 2 G Trap Column (180 µm × 20 mm, 5 µm) and separated on a nanoAcquity UPLC Peptide BEH C18 column (75 µm × 100 mm, 1.7 µm). All raw data files were analyzed using Thermo Scientific Proteome Discoverer 3.0 software and protein profiles were searched in the UniProt *Lm* EGD database. The peak intensity of each protein was quantified, and FDR at the protein level was <1%. A pseudocount was used, adding 1 to all peak intensity values. Genes with ± 1 logFC (stress/normal peak intensity) were classified as up- or down-regulated; proteins not meeting this threshold were considered as non-significant (NS). Statistical significance was assessed by two-sample t-tests (*P* *<* 0.05). Volcano plots were generated using -log_10_ (*P*-value).

### Scanning electron microscopy (SEM)

The pellets of SR-*Lm* in (1) normal (37 °C), (2) 5% salt+1 °C, (3) pH 3 + 1 °C, and (4) pH 3 + 1 °C + 5% salt at 12 h was prepared by washing with phosphate-buffered saline (PBS, Invitrogen, Waltham, MA, USA) and fixed in 2.5% glutaraldehyde in 1× PBS. A 0.4 µm Nucleopore Track-Etched Membrane was wetted with water and placed in a 13 mm Swinnex filter holder. Rehydration was performed through a graded series of ethanol (30–100%). Critical point drying was conducted using a CO_2_ critical point dryer (Electron Microscopy Sciences, Hatfield, PA, USA). The dried samples were mounted on aluminum stubs and sputter-coated with a thin layer of gold-palladium to enhance conductivity. The samples were imaged using a dual-beam SEM equipped with a focused ion beam (FIB) on a FEI Quanta 3D SEM (Thermo Fisher Scientific) at 10 keV.

### Data analysis

All statistical methods including two-way ANOVA for death curves and differentially expressed genes and proteins were analyzed in R Studio (version 4.3.3, PBC, Boston, MA, USA). Gene Ontology (GO) enrichment was conducted using upregulated and downregulated genes and proteins at https://geneontology.org/. KEGG (Kyoto Encyclopedia of Genes and Genomes) pathways in each stress condition were predicted using proteomic data at https://www.kegg.jp/.

## Results

### Death curves

**Figure 1:**
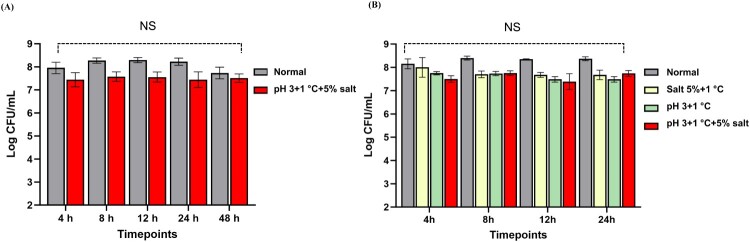
. (A) Death curves for RNAseq of SR*-Lm* under normal and multiple stress conditions over 48 h, showing viable cell counts. (B) Death curves for proteomic analysis of SR*-Lm* under normal and three stress conditions: 5% salt+1 °C, pH 3 + 1 °C, and pH 3 + 1 °C + 5% salt. Non-significant values are indicated as NS.

Figure 1A shows the survival curves of SR*-Lm* under normal and multiple stress conditions. During 48 h incubation, viable cell counts of *Lm* under stress conditions decreased by less than 1 log CFU/mL and were not significantly different compared to the controls. The death curve of proteomic analysis was conducted under normal and three stress conditions (Figure 1B). Viable cell counts of the SR-*Lm* were approximately 7 to 8.5 log CFU/mL under the four conditions for 24 h incubation. No significant differences in viable cell counts were observed across time points or conditions.

### Transcriptomic analysis (RNAseq)

**Figure 2.**
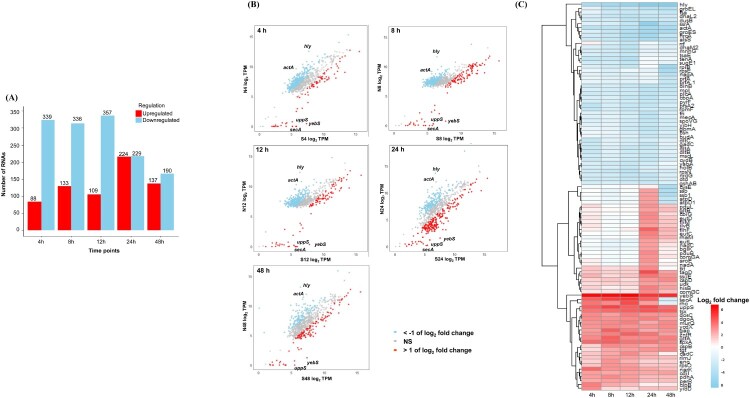
(A) Number of upregulated and downregulated genes. (B) RNA expression patterns at five time points. (C) Heatmap displaying the top 50 upregulated and bottom 50 downregulated genes. All transcriptomic analyses of SR-*Lm* were conducted under multiple stress conditions (pH 3 + 1 °C + 5% salt) compared to normal conditions for 48 h. A cutoff of ±1 logFC was used to classify upregulated (red), downregulated (blue), and non-significant (gray) genes.

A total of 751 transcripts were identified, of which 689 mRNAs, 4 ncRNAs, 57 rRNAs, and one tRNA were determined (Table S1). The number of differentially expressed transcripts showed a pattern similar to a prior study [9], ranging from 88–224 upregulated and 190–357 downregulated over 48 h (Figure 2A). Scatterplots of SR-*Lm* for 48 h are shown in Figure 2B (cutoff = 1.0). The 8 and 12 h showed similar gene expression patterns, whereas the 24 h showed many upregulated transcripts. The heatmaps (Figure 2C and Figure S1) clearly show that *yebS, secA, tsx, gap,* and *ycdX* genes were overexpressed under multiple stress conditions at 48 h. Genes associated with acid, high salt, and low temperature stress are presented in Table S2, as reported in previous studies [7,15–17]. The *spxA* (salt resistance) and *cspB*/*cspD* (cold shock) were upregulated from 4 to 48 h. The *atpD* (acid resistance) peaked at 24 h, and *tkt* (metabolism) was upregulated from 4 to 24 h. The upregulation patterns to stress (*spxA* and *cspD*) were consistent with their established roles in stress response (salt and cold stress) [18,19]. RT-qPCR validation was performed for six genes across five time points (n = 30). Regression analysis revealed a strong correlation between RT-qPCR assay and RNAseq results (R² =  0.748) (Figure S2, Table S3). This validation is more comprehensive than previous studies, which typically validate only 1–4 genes, providing stronger confidence in the RNAseq data in this study [18,20].

### Protein expression analysis

**Figure 3.**
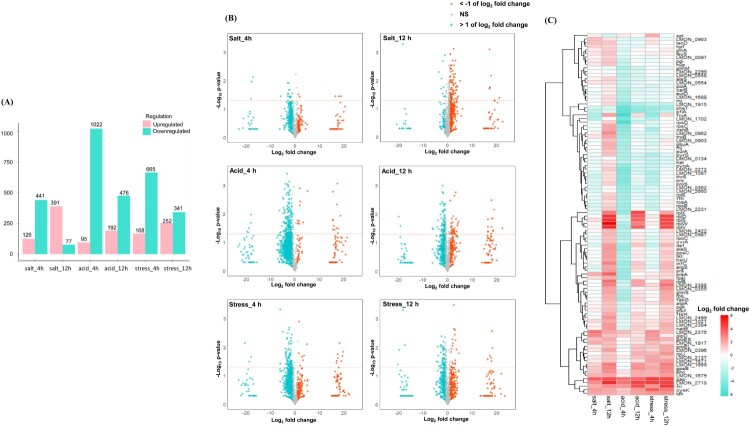
(A) Number of regulated proteins: The bar graph shows the count of upregulated (red) and downregulated (green) proteins (B) Volcano plot: The x-axis represents logFC (stress/normal peak intensity) and the y-axis shows statistical significance (-log10 *P*-value) from a two-sample t-test. (C) Heatmap: Displays the top 50 upregulated and bottom 50 downregulated proteins. All proteomic analyses were conducted under three stress conditions using a logFC cutoff of ±1 (stress/normal peak intensity) and statistical significance of *P* < 0.05.

A total of 1,034 proteins were identified (Table S4) in normal, acidic, high salt, and multiple stress conditions. The number of differentially expressed proteins ranged from 95 to 391 upregulated and from 77 to 1022 downregulated (Figure 3A). As shown in the volcano plots (Figure 3B), downregulated proteins were predominantly observed under acid and multiple stress conditions at 4 h, while upregulated proteins were detected under all three conditions at 12 h. Heatmaps based on the logFC and *P*-values are shown in Figure 3C (top/bottom 50) and Figure S3. At 12 h, the proteins RplD and RplV (ribosomal proteins) were upregulated, suggesting that the structural stability of ribosomes in *Lm* exposed to stress might facilitate the translation of mRNA into proteins [15]. Proteins Gap, Fri, Eno, and CysK were consistently upregulated regardless of the time point and stressors.

### Gene ontology & KEGG pathway

**Figure 4.**
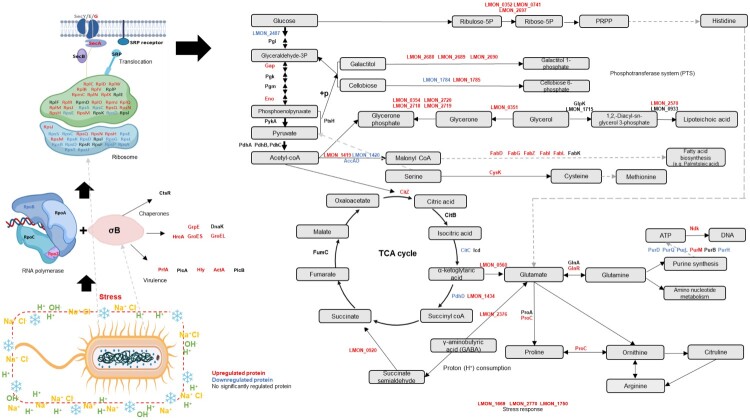
KEGG pathway of SR-*Lm* using proteins under multiple stress conditions (pH 3 + 1 °C + 5% salt). Proteins upregulated at any single time point (4 or 12 h) are shown in red (upregulated), while those consistently downregulated at both 4 and 12 h are shown in blue (downregulated). Non-significant proteins are shown in black. Black arrows represent the normal pathway, while gray arrows indicate distant alternative pathways.

GO analysis using up- and down-regulated RNA and proteins was conducted (Table S5–S8). Among the upregulated categories, those involved in oxidoreductase activity, or ATPase-related activities, were significantly upregulated in both analyses. GO terms of DNA damage response, replication, and recombination were consistently downregulated across both analyses. The KEGG pathway was integrated from both 4 and 12 h time points to capture the complete temporal protein profiles (early and late response). The pathway analysis for SR-*Lm* under multiple stress conditions based on proteomic analysis is presented in Figure 4 [16]. Consistent with previous studies, σB appeared to be activated in response to stress, initiating the regulation of chaperones (GrpE, GroES, and GroEL) and *Listeria* virulence (PrfA) [17,21]. Upregulated ribosomal proteins (RplC, RplD, and RplW) supported the activation of proteins associated with stress resistance. Proteins involved in histidine biosynthesis, PTS for sugar uptake (galactitol), GAD, and parts of lipoteichoic acid (cell wall) and fatty acid biosynthesis were upregulated. In Figure S4, the salt stress pathway demonstrated upregulation of PTS for fructose, osmoprotectants (GbuAB and OpuCAD), and glycine and heme-related proteins (HemC and LMON_0889). Under acid stress, the downregulated proteins in the pathway were observed, including those associated with chaperones (HrcA), lipoteichoic acid, and fatty acid biosynthesis (Figure S5).

### Cell morphology

**Figure 5.**
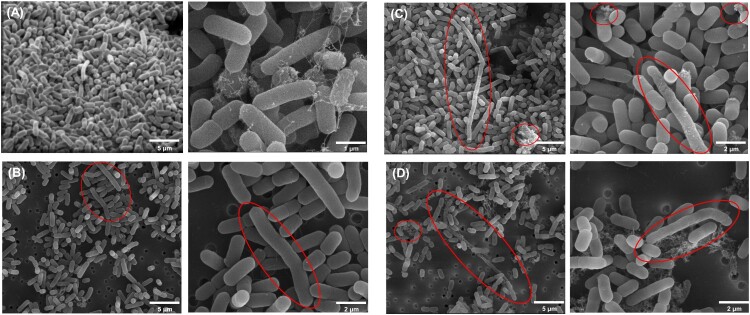
SEM images of SR*-Lm* after 12 h of incubation under (A) Normal, (B) 5% salt+1 °C, (C) pH 3 + 1 °C, and (D) pH 3 + 1 °C + 5% salt. Red circles highlight key features of interest in each image.

The cell morphology of *Lm* under normal and three different stress conditions at 12 h was captured (Figure 5). In normal conditions, the cell morphology displayed extensive clustering and biofilm formation. Cells under the salt stress condition exhibited similarities to those under normal conditions. Under acid and multiple stress conditions, elongated shapes were specifically predominant. Small clusters observed under these conditions suggested extracellular polymeric substances (EPS) with the cells exhibiting skinny, bumpy, and elongated or damaged cells [22]. This morphological change was accompanied by the upregulation of cell shape protein MreC, peptidoglycan synthesis proteins (MurB and MurF), and membrane-associated proteins (RacE and LMON_1400). However, these proteins (e.g. MurB and MurF) were either non-significant or downregulated under acid or multiple stress conditions.

## Discussion

The tendency toward non-significant changes in viable cell counts was consistent between previous studies and the current study [7,8]. The transcriptomic analysis revealed upregulation of the *yebS* gene, which encodes an inner membrane transport protein related to an oxidative stress response [23]. The *tsx* gene encodes a nucleoside-specific channel-forming protein in the outer membrane of Gram-negative bacteria. Previously, *tsx* expression in *Salmonella enterica* increased under anaerobic conditions, leading to improved survival in macrophages [24]. However, *tsx* gene upregulation has not been reported in recent transcriptomic studies of *Lm*. The overexpressed *secA,* involved in the process of protein transport, was evident (8–24 h) in both transcriptomic and proteomic analyses. This *secA* might be indirectly affected by the stress response, potentially enhancing correct protein translocation during synthesis and improving protein folding and transport under stress conditions. Both *gap* and *gtfA* genes are involved in the energy-associated pathway in *Lm.* Therefore, SR-*Lm* may require increased energy production and consumption to enhance the ability to regulate pH homeostasis for protection under multiple stress conditions, leading to the upregulation of this gene in this study [25,26].

Previous studies have examined factors such as the σB (*sigB*), stressosome, virulence connection, and surface-dependent responses to the stress response of *Lm* [19,27]. Several related genes were observed in the present study, including *rpoN*, *sigB*, *tkt*, and *ptsP*. The expression level of the *sigB* gene slightly increased in the transcript expression over 48 h incubation with some fluctuation and showed an increase after 12 h in the proteomic analysis. The response of *sigB* to stress, particularly acid stress, was consistent with findings from previous studies [28,29]. In stress-related genes, *cysK* (cysteine synthase) gene was upregulated in both transcriptomic and proteomic analyses under all stress conditions. Although cysteine is known as a general metabolism protein in *Lm*, it may also serve as a potential general stress response protein in *Lm* [8]. The *trxA* (thioredoxin) gene showed upregulation in transcriptomic analysis at all time points. Both *cysK* and *trxA* are involved in oxidative stress responses in bacteria [30], suggesting that these genes help maintain cellular redox homeostasis during stress.

The tryptic soy broth used in this study comprises nutrients such as glucose, dextrose, and casein, and this composition is speculated to predict the KEGG pathways (Figure 4). Once *Lm* detects stress, σB initially plays a role in the multiple stress response by binding to RNA polymerase (e.g. RpoZ) [19,21]. The σB-polymerase complex (e.g. RpoA) transcribes stress-related genes, while upregulated ribosomal proteins (e.g. RplC and RplD) facilitate mRNA translation into proteins. Chaperones (e.g. HrcA) ensured correct protein folding, and translocation proteins (e.g. SecA) directed synthesized proteins to their functional locations [31]. Metabolic pathways are reprogrammed for energy production. Carbon metabolism and PTS systems (e.g. LMON_2487 and 2688) and cellobiose metabolism (LMON_1784 and 1785) were upregulated, likely enabling the utilization of alternative carbon sources when primary nutrients are limited. Other assumptions include ATP production, proton consumption, ammonia neutralization via proton exchange, and the uncharacterized stress response proteins (LMON_1669 and LMON_2770) [18,26]. Some lipoteichoic acid and fatty acid proteins (e.g. LMON_2718 and FadG) showed upregulation, indicating the cell envelope remodeling in membrane adaptation, repair, or unsaturated fatty acid production [32].

Similarly, the proteomic profiles under the salt stress condition closely resembled that observed under multiple stress conditions (Figure S3). The upregulated proteins at 12 h were more broadly distributed under salt stress compared to those under acidic and multiple stress conditions. Unique upregulated proteins in salt stress were associated with the PTS system for fructose/fructose-1,6-bisphosphate, glycine-serine-tryptophan, cysteine-methionine metabolism, and osmoprotectant transport systems (GbuABC and OpuCAD). These processes are involved in sugar/carbohydrate uptake for energy production or oxidative stress protection [6,33]. Notably, the upregulation of osmolyte transport systems (GbuAB, OpuCA, and OpuCD) was consistent with previous findings to emphasize compatible solute roles to salt stress [6]. These results emphasized that the osmolyte transport systems are strongly involved in salt stress adaptation mechanisms of *Lm*.

On the other hand, upregulated proteins under acid stress in the pathway included histidine biosynthesis, galactitol metabolism, and virulence factors PlcA and PlcB (Figure S5). These were associated with inositol phosphate metabolism and glycerophospholipid biosynthesis, which are linked to fatty acid metabolism, and most importantly, facilitate virulence and intracellular survival. Despite these differences in proteomic profiles, the viable cell counts under acid stress were comparable to those observed under salt and multiple stress conditions, highlighting the remarkable resilience of *Lm* to acid stress. This may be attributed to cross-protection, a phenomenon where prior exposure to one type of stress enhances survival against other stresses [34]. Notably, histidine biosynthesis-associated proteins (e.g. LMON_2697) were upregulated under all three stress conditions. While a prior study demonstrated that histidine transport aids *Staphylococcus* in acid stress resistance [35], our finding of histidine biosynthesis upregulation in *Lm* represents a novel stress response mechanism. On the other hand, the GAD systems (LMON_2376) were upregulated under multiple and high salt stress. The GAD system is a well-established acid stress resistance [6] that consumes intracellular H^+^ during glutamate-to-GABA conversion, thereby neutralizing intracellular acidity and maintaining pH homeostasis [36]. Additionally, succinate derived from GABA metabolism enters the TCA cycle and produces energy, GTP via substrate-level phosphorylation. This represents a metabolic reprogramming strategy where cells optimize energy production efficiency to survive stressors. In this study, the multiple stress condition likely triggered a more comprehensive stress response, potentially through synergistic effects or a more energy-efficient survival mechanism under nutrient-limiting conditions [37]. These findings suggest that *Lm* prioritizes specific pathways to multiple stresses, optimizing resource allocation for survival rather than activating acid-specific stress responses alone.

Key GO terms were identified with proteins in the KEGG pathways, including oxidoreductase activity, protein-containing complex, electron transport chain, ribosomal, DNA damage, and chaperone functions [26,38]. These GO terms suggest that chaperones reorganize in protein folding and stability including in acid tolerance factors, vital for maintaining cellular integrity during stress [38]. For instance, the electron transport chain and oxidoreductase activities are essential for managing reactive oxygen species (ROS) levels, while ribosomal genes enhance protein synthesis, thereby supporting cellular functions under stress [26,38–40]. This interplay between these functions helps *Lm* survive and adapt to challenging environments, highlighting the importance of gene regulation in stress responses [41]. In addition, the ATP-related GOs (e.g. ATP synthesis coupled electron transport) were significantly upregulated in both analyses, consistent with previous studies in bacteria [9,42]. This result may explain the downregulation of genes involved in GO DNA damage response, suggesting that before DNA damage occurs due to stress, SR*-Lm* enhances its ABC transporter or energy metabolism to respond to stress conditions. On the other hand, some GO terms such as carboxylic acid biosynthetic process and aromatic amino acid metabolic process were not consistent in both analyses. Observed discrepancies between transcript and protein levels likely reflect post-transcriptional regulatory mechanisms (translational efficiency, ribosome binding, and translation initiation rates), and methodological differences [43,44].

Several studies have reported elongated cells of SR-*Lm* in diverse environments. Previously, cell elongation was observed in *Lm* under pH 9.0 or high salt environments (12.5%) [45,46]. This elongation indicated that pH influences cell shape and involves stress adaptation mechanisms [45,46]. Similarly, *Lm* exposed to chlorine-induced oxidative stress also exhibited cell elongation, which correlated with DNA damage and upregulation of recombination, repair, and error-prone polymerases for stress adaptation [47,48]. Regarding the rough cell surface, *Lm* treated with nodule-specific cysteine-rich peptides developed a rough surface, suggesting that the antimicrobial activity was linked to membrane permeabilization and the release of cellular content [49]. In this present study, cell shape and peptidoglycan proteins MreC, MurB, and LMON_1501 were only upregulated under salt stress conditions. These proteins may contribute to maintaining a cell shape in salt stress, similar to normal conditions, potentially explaining why some cells had elongated and bumpy morphologies. MreB and MreC proteins, actin-related, energy-dependent proteins involved in maintaining cylindrical cell shape in bacteria, were downregulated [50]. This downregulation of cell shape-related genes in stress environments accounts for the observed morphological changes. PbpB was upregulated at 12 h under all stress conditions, suggesting a role in maintaining cell wall integrity and synthesis rather than influencing cell shape. Small clusters observed under acid and multiple stress conditions might represent biofilms composed of extracellular polymeric substances influenced by stress [22]. These biofilms are known to enhance virulence and survival or protection strategies under stress [51]. Bacterial cells typically reach a certain size before initiating DNA replication [52]. During this process, nutrient availability and environmental factors influence the timing of DNA replication and cell division [52]. In DNA replication, the DNA strands separate, allowing DNA polymerase to synthesize new strands bidirectionally. This process highlights the significant impact of DNA replication on cell division [52]. When *Lm* was exposed to stress, the cells might attempt to replicate their DNA or grow. However, under stress conditions, associated genes might be damaged, potentially leading to abnormal cell morphologies [53]. This observation aligned with our analyses, including GO analysis, where DNA replication (e.g. *dnaB*) was downregulated in three stress conditions.

The current study has provided comprehensive insights into the stress response mechanisms of SR*-Lm*. The stress response mechanisms were predicted including energy production, proton consumption, ammonia neutralization, and stronger cell wall components. Cell shape proteins were significantly downregulated under acid and multiple stress conditions, whereas they remained unchanged under salt stress, suggesting cell shape genes may contribute to altered cell morphology. The present research is the first omics-based study of SR*-Lm* and provides a foundation for understanding pathogens at the molecular level.

## Supplementary Material

Supplemental Material

Supplemental Material

TableS4_revised.xlsx

Supplementary_Figure_1_revised.pdf

Supplementary_Figures_3_revised.pdf

TableS5_revised.xlsx

TableS1.xlsx

TableS2_revised.xlsx

TableS8_revised.xlsx

TableS3_revised.xlsx

TableS6_revised.xlsx

TableS7_revised.xlsx

Supplementary_Figures_2_revised.pdf

## Data Availability

The whole transcriptome sequences in this study are available at the GenBank of the National Center for Biotechnology Information (NCBI): PRJNA1148455.
