# Supplementary material for: New insights into the stress response mechanisms of stress-resistant Listeria monocytogenes via multi-omics and cell morphological changes
Source: Emerg Microbes Infect. 2025 Sep 19;14(1):2564319. doi: 10.1080/22221751.2025.2564319 (PMC12498373; doi:10.1080/22221751.2025.2564319)

Supplementary Figure 5. . KEGG pathway of stress-resistant *L. monocytogenes* using proteins under acid stress conditions (pH 3+1 °C). Proteins upregulated at any single time point (4 or 12 h) are shown in red (upregulated), while those consistently downregulated at both 4 and 12 h are shown in blue (downregulated). Proteins with log_2 fold changes between -1 and 1 (non-significant) are shown in black. Background proteins are shown in gray, and important processes or products are highlighted in green. Black arrows represent the normal pathway, while gray arrows indicate normal or distant alternative pathways. PRPP: phosphoribosyl pyrophosphate.

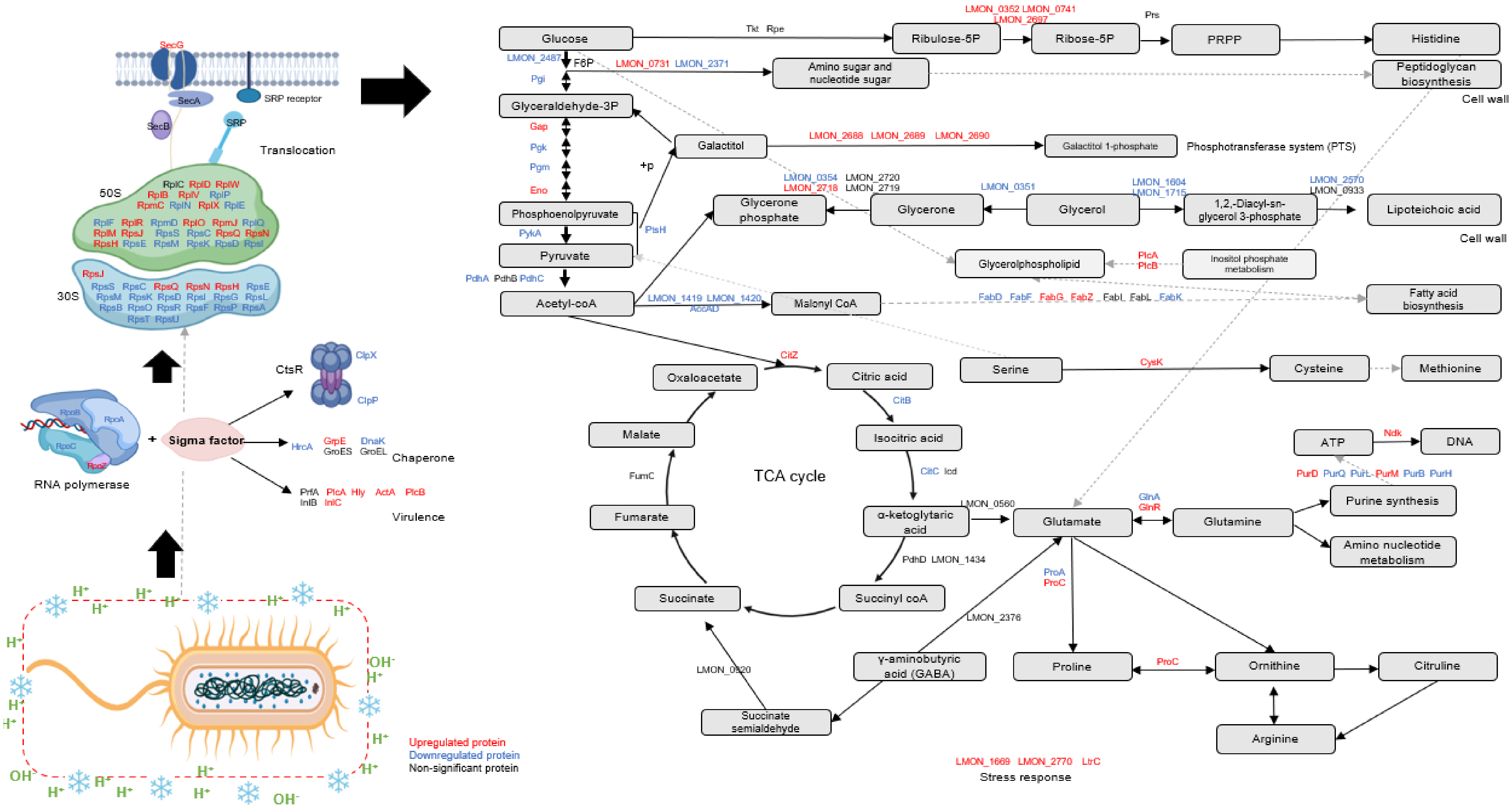

Supplement: Supplemental Material [file TEMI_A_2564319_SM6140.pdf]
