# Supplementary material for: New insights into the stress response mechanisms of stress-resistant Listeria monocytogenes via multi-omics and cell morphological changes
Source: Emerg Microbes Infect. 2025 Sep 19;14(1):2564319. doi: 10.1080/22221751.2025.2564319 (PMC12498373; doi:10.1080/22221751.2025.2564319)

Supplementary Figure 1. (A) Heatmap displaying the top 100 and (B) bottom 100 gene expression patterns (RNA) of stress-resistant L. monocytogenes under multiple stress conditions (pH 3+1 °C+5% salt) for 48 h, compared to normal conditions, using log_2 fold change (stress/normal TPM). In both heatmaps, log_2 fold change (fc = stress TPM/normal TPM) was used as the expression level. Genes with a log_2 fold change greater than 1.0 are shown in red (up-regulated), those with a log_2 fold change less than -1.0 are shown in blue (down-regulated), and genes with log_2 fold changes between -1.0 and 1.0 are shown in gray, indicating no substantial change (non-significant).

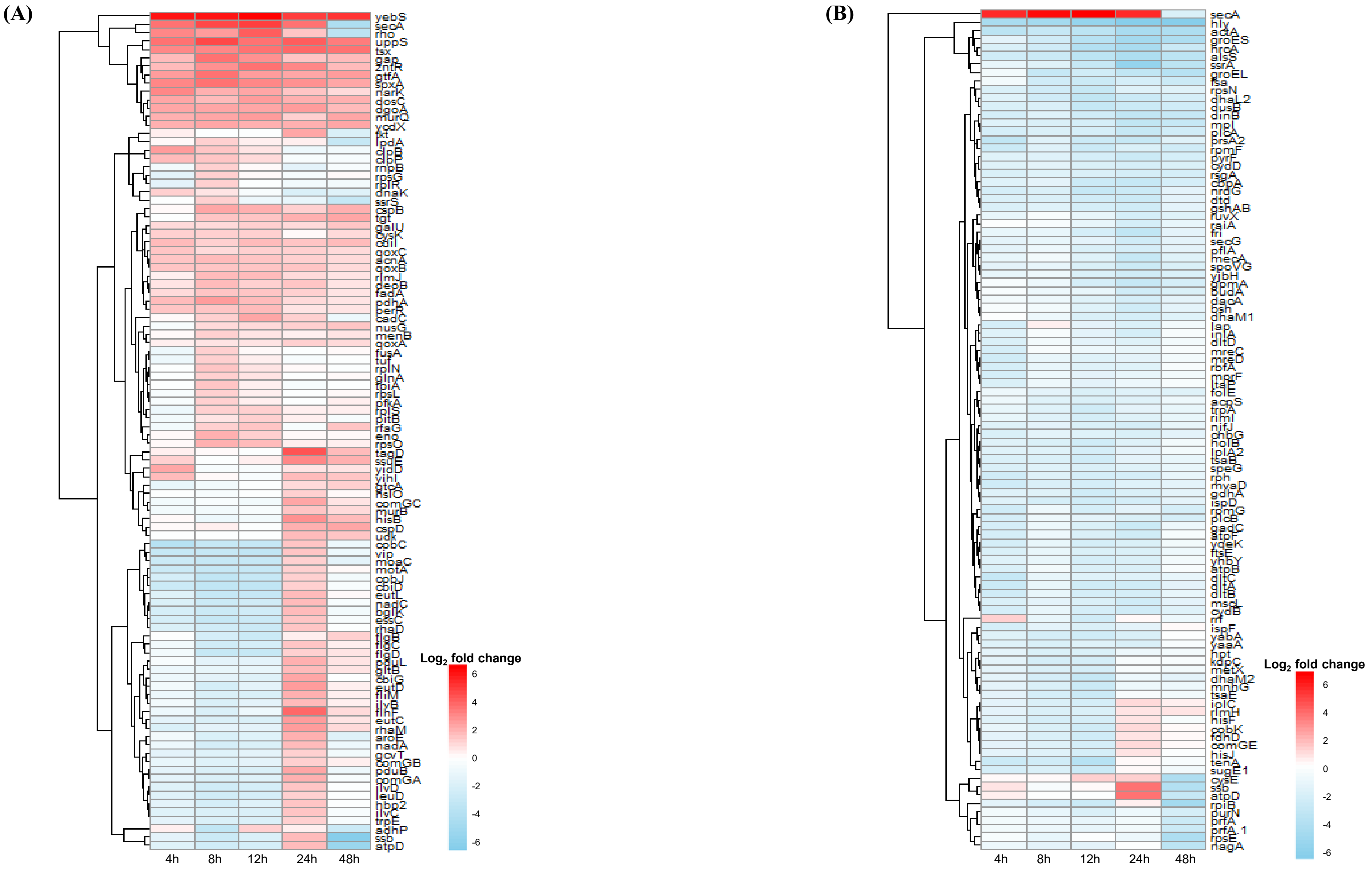

Supplement: Supplementary_Figure_1_revised.pdf [file TEMI_A_2564319_SM3252.pdf]
