# Supplementary material for: New insights into the stress response mechanisms of stress-resistant Listeria monocytogenes via multi-omics and cell morphological changes
Source: Emerg Microbes Infect. 2025 Sep 19;14(1):2564319. doi: 10.1080/22221751.2025.2564319 (PMC12498373; doi:10.1080/22221751.2025.2564319)

Supplementary Figure 3. (A) Heatmap displaying the top 100 and (B) bottom 100 proteins of stress-resistant *L. monocytogenes* under different stress conditions compared to normal conditions, using $\log_2$ fold change (stress/normal peak intensity). Proteins with significant values were selected for this analysis. (C) Heatmaps of the top 50 and bottom 50 proteins under independent stress conditions, regardless of significance (p-value). In all heatmaps, $\log_2$ fold change (fc = peak intensity under stress/normal) was used as the protein expression level. Proteins with a $\log_2$ fold change greater than 1.0 are shown in red (up-regulated), those with a $\log_2$ fold change less than -1.0 are shown in blue (down-regulated), and proteins with $\log_2$ fold changes between -1.0 and 1.0 are shown in gray, indicating no substantial change (non-significant).

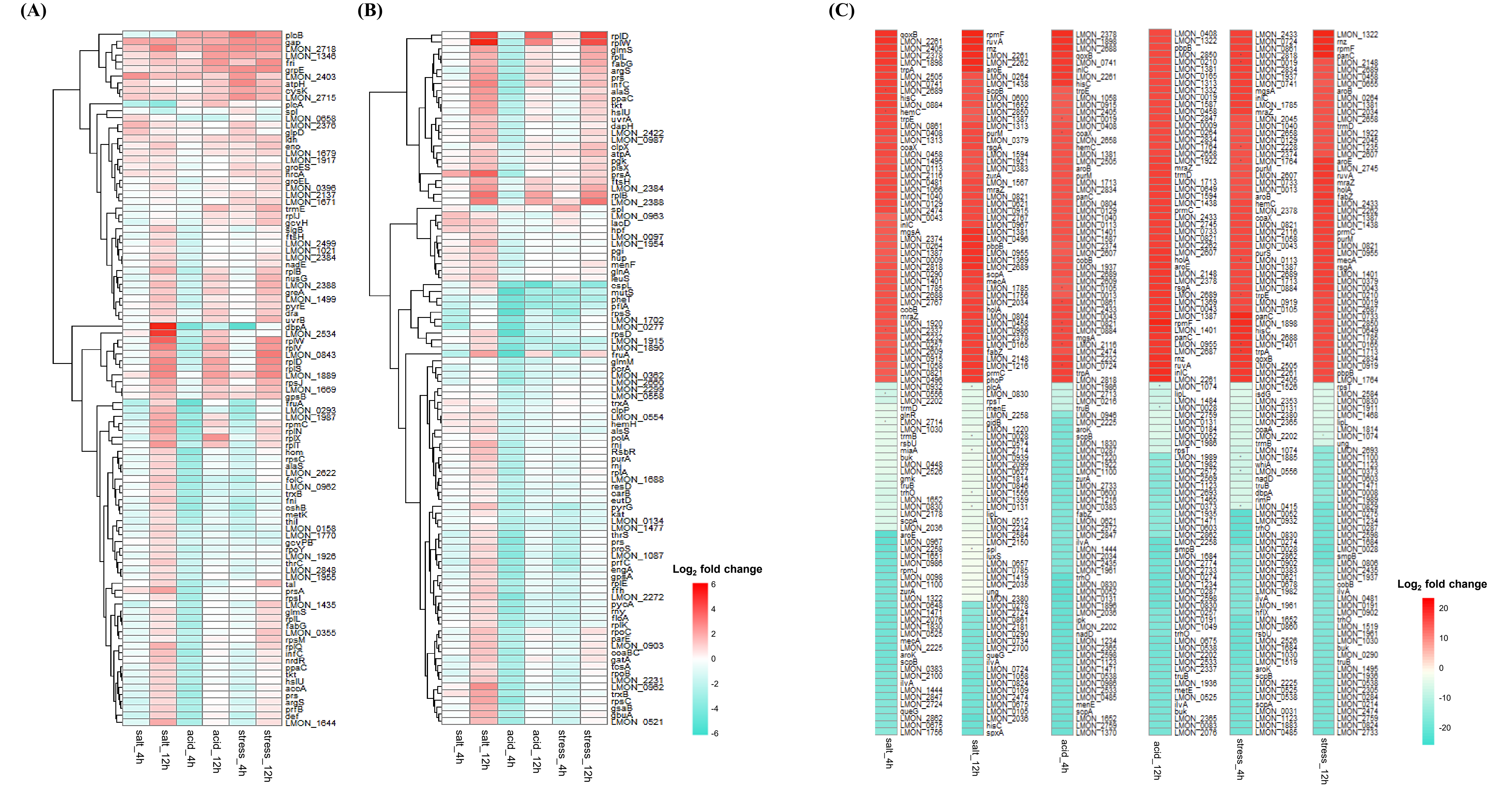

Supplement: Supplementary_Figures_3_revised.pdf [file TEMI_A_2564319_SM3251.pdf]
