# Supplementary material for: New insights into the stress response mechanisms of stress-resistant Listeria monocytogenes via multi-omics and cell morphological changes
Source: Emerg Microbes Infect. 2025 Sep 19;14(1):2564319. doi: 10.1080/22221751.2025.2564319 (PMC12498373; doi:10.1080/22221751.2025.2564319)

Supplementary Figure 2. Validation of RT-qPCR and RNAseq using the 2-ΔΔCT method, presented as logFC for each method. Primer information is available on Supplementary Table 8.

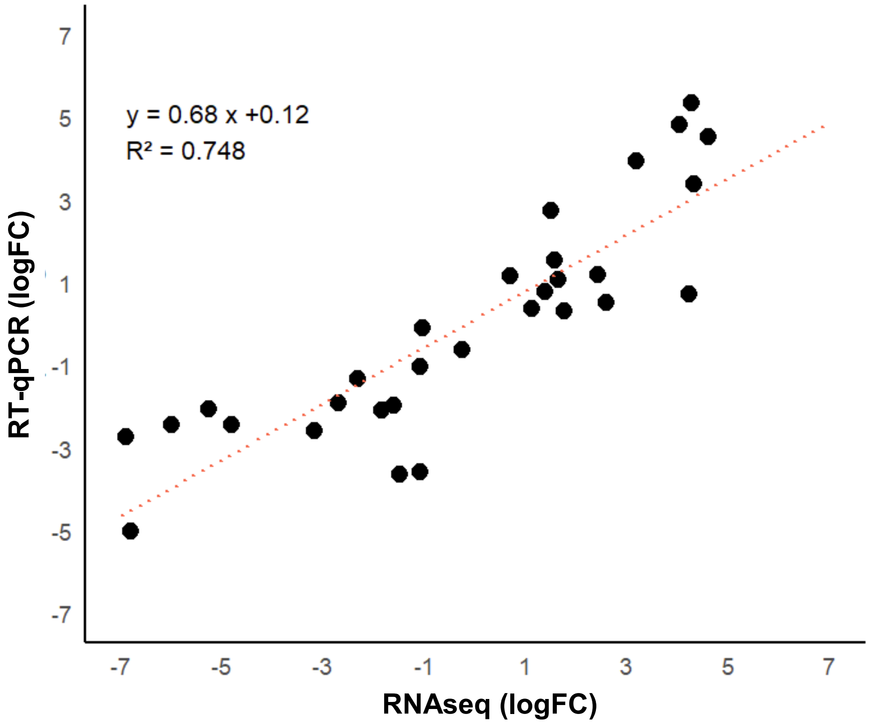

Supplement: Supplementary_Figures_2_revised.pdf [file TEMI_A_2564319_SM3242.pdf]
